# Analysis of factors influencing kinesiophobia in post-cardiac surgery patients and construction of a nomogram prediction model

**DOI:** 10.1186/s12872-026-06301-x

**Published:** 2026-08-01

**Authors:** Zihan Hong, Mengjun Zhan, Yao Shi, Junjun Gu

**Affiliations:** Cardiac Surgery Intensive Care Unit, The First Affiliated Hospital of Naval Medical University, Shanghai, 200433 China

**Keywords:** Post-cardiac surgery, Kinesiophobia, Risk prediction model, Nomogram

## Abstract

**Background:**

A nomogram model was developed to assess the risk of kinesiophobia in post-cardiac surgery patients, aiming to provide a basis for the early identification of high-risk individuals and guide clinical interventions.

**Methods:**

This study employed a cross-sectional design and recruited post-cardiac surgery patients admitted to a tertiary Grade A hospital in Shanghai between June 2024 and June 2025. Univariate analysis and logistic regression analysis were performed using SPSS 27.0 to identify independent risk factors for kinesiophobia. Internal validation of the model was carried out in R 4.4.3 using the Bootstrap method with 1,000 resamples. The predictive performance of the model was assessed using the area under the receiver operating characteristic curve (AUC), calibration curves, and decision curve analysis (DCA), and a nomogram was generated to visually represent the model.

**Results:**

A total of 477 valid questionnaires were collected, among which 292 patients exhibited kinesiophobia, corresponding to a prevalence of 61.22%. Logistic regression analysis indicated that pain intensity, social support level, fatigue, Type D personality, and educational level were independent risk factors for kinesiophobia in post-cardiac surgery patients (*P* < 0.05). The calibration curve, receiver operating characteristic (ROC) curve, and DCA all demonstrated the model’s high quality.

**Conclusion:**

Post-cardiac surgery patients are at a high risk of developing kinesiophobia. The nomogram model developed in this study can provide healthcare professionals with a practical tool for the early identification of high-risk individuals and the implementation of targeted interventions.

**Supplementary Information:**

The online version contains supplementary material available at https://doi.org/10.1186/s12872-026-06301-x.

## Introduction

Cardiovascular disease (CVD) represents a major global public health challenge. According to findings from the global burden of disease study [1, 2], CVD has consistently ranked among the leading causes of death worldwide, and its morbidity and mortality burden continues to increase [3]. For patients whose conditions cannot be adequately controlled with pharmacological therapy or who present with severe structural heart disease, cardiac surgery remains an important therapeutic approach for alleviating symptoms, prolonging survival, and improving quality of life [4]. With continuous advances in surgical techniques, cardiopulmonary bypass management, and perioperative care, perioperative safety and short-term survival outcomes in cardiac surgery patients have improved substantially. Nevertheless, inadequate postoperative functional recovery, poor exercise adherence, and differences in long-term prognosis remain significant clinical challenges.

Cardiac rehabilitation, with exercise training as its core component, has been recognized as an essential part of recovery for patients following cardiac surgery [5, 6]. Regular and progressive exercise training can improve left ventricular ejection fraction, reduce the risk of adverse cardiovascular events, decrease hospital readmission rates, and enhance psychological well-being and quality of life [7, 8]. However, despite the well-established benefits of exercise-based rehabilitation, some postoperative patients develop marked avoidance of and fear toward physical activity because of concerns that exercise may induce chest pain, wound disruption, cardiac events, or physical dysfunction. This psychological phenomenon is commonly referred to as kinesiophobia [9]. Kinesiophobia was initially reported primarily in patients with chronic pain [10] and those recovering from orthopedic surgery [11], but increasing attention has recently been directed toward this condition in patients with cardiovascular disease and those undergoing cardiac rehabilitation. Previous studies have shown that [12] participation in exercise rehabilitation among post-cardiac surgery patients is generally low, and that 39.20%–82.57% of these patients experience kinesiophobia. This psychological state may reduce adherence to cardiac rehabilitation exercise programs, resulting in progressive declines in postoperative aerobic capacity and muscle strength, thereby increasing the risk of recurrent cardiovascular events and delaying the overall recovery process [13, 14].

Compared with other postoperative populations, post-cardiac surgery patients exhibit more distinct disease-specific characteristics and unique rehabilitation needs. On the one hand, cardiac surgery is highly invasive. In particular, following median sternotomy, patients often experience sternal incision pain, concerns regarding upper-limb movement, and anxiety about cardiac safety [15]. On the other hand, patients frequently experience anxiety, depression, and sleep disturbances, all of which may collectively contribute to the development or exacerbation of kinesiophobia [16, 17]. Therefore, the mechanisms underlying kinesiophobia in post-cardiac surgery patients may differ from those in patients undergoing orthopedic surgery or those with chronic pain conditions. Consequently, assessment methods and intervention strategies developed for other populations cannot be directly applied to this patient group.

Compared with previous domestic and international studies, the prevalence of kinesiophobia among post-cardiac surgery patients remains relatively high, suggesting that this issue is common across different regions and healthcare systems. However, existing studies have mainly focused on the prevalence of postoperative kinesiophobia and the analysis of individual influencing factors. To date, no assessment tool specifically designed for post-cardiac surgery settings with good interpretability and clinical applicability has been established. Consequently, accurately identifying high-risk patients and implementing stratified interventions during early hospitalization or before discharge remains challenging in clinical practice.

In recent years, the deep integration of artificial intelligence technologies with clinical big data has provided new opportunities for personalized risk assessment. By integrating multidimensional influencing factors, risk prediction models can quantitatively estimate the probability of disease occurrence or adverse outcomes, thereby providing a reliable basis for clinical decision-making [18]. As an intuitive, quantitative, and clinically applicable predictive tool, nomograms have been widely used in risk assessment for various diseases.

Based on the above considerations, this study aimed to analyze the factors associated with kinesiophobia in post-cardiac surgery patients and to further develop and validate a nomogram prediction model. The findings are expected to provide a basis for the early identification of high-risk patients, the implementation of individualized interventions, and the optimization of cardiac rehabilitation management.

## Methods

### Study design

This study employed a cross-sectional design and included post-cardiac surgery patients receiving care at the First Affiliated Hospital of the Naval Medical University. The study strictly adhered to the ethical principles of the Declaration of Helsinki [19], ensuring that participants’ rights were fully protected and that no risk or harm was posed to them. The study was conducted following review and approval by the hospital’s Institutional Review Board (IRB) (Ethics Approval Number: CHEC2018-136).

### Sample size calculation

Within the framework of a cross-sectional epidemiological study, the sample size was calculated using a standard sample size formula [20]: $n=\frac{Z_{1-\alpha/2}^{2}\cdot{P}\cdot(1-P)}{d^{2}}$. Based on a preliminary survey, the risk of kinesiophobia among post-cardiac surgery patients was estimated at 65% (*P* = 0.65). The margin of error (d) was set at 5%, and a 95% confidence interval (CI) with a two-tailed test was assumed, yielding Z_1−α/2_=1.96. In addition, to ensure the adequacy of the final sample, a 10% attrition rate was taken into consideration. Accordingly, the minimum required sample size for this study was 389 patients.

### Study population

The study employed convenience sampling to recruit post-cardiac surgery patients treated at the First Affiliated Hospital of the Naval Medical University between June 2024 and June 2025. Inclusion criteria were: (1) Age ≥ 18 years; (2) Underwent cardiac surgery at this hospital, with a stable postoperative condition and adequate mobility; (3) Clear mental status, unobstructed verbal communication, and the ability to cooperate in completing the questionnaire; (4) Voluntary participation and provision of written informed consent. Exclusion criteria were: (1) Severe concomitant hepatic or renal insufficiency, malignant tumors, severe primary neurological or musculoskeletal diseases, or severe organ failure; (2) History of psychiatric disorders or ongoing psychiatric medication; (3) Contraindications to physical activity according to expert consensus [21], including unstable angina, acute heart failure, or recent extensive myocardial infarction, as well as other organic conditions causing significant mobility impairment, such as venous thrombosis or lower limb fractures. Additional elimination criteria included: (1) Participants with a questionnaire missing rate > 10%; (2) Significant deterioration of data quality during the survey due to emotional instability or poor cooperation; (3) Voluntary withdrawal from the study or sudden deterioration of clinical condition.

### Research tools

#### Variable assessment tools

1. Social Support Rating Scale (SSRS): The SSRS comprises 10 items across three dimensions: objective support, utilization of social support, and subjective support [22]. The total score ranges from 12 to 66, with the objective support dimension scoring 4–16, the subjective support dimension scoring 5–38, and the utilization of support dimension scoring 3–12; higher scores indicate greater perceived social support. Based on the total score, social support can be classified into three levels: low (12–22), medium (23–44), and high (45–66). The SSRS has demonstrated good reliability and validity, with a Cronbach’s α coefficient of 0.91 [22];
2. Barthel Index (BI): The BI comprises 10 items assessing core activities of daily living, including eating, personal hygiene, dressing, toileting, mobility, and walking. Each item is scored according to the patient’s ability to perform the task independently. Total scores range from 0 to 100, with higher scores indicating greater independence in daily activities. The scale has demonstrated good reliability, with a Cronbach’s α coefficient of 0.883 [23];
3. Fatigue Severity Scale (FSS): This scale consists of 9 items and uses a 7-point Likert scale, ranging from 1 (“Strongly Disagree”) to 7 (“Strongly Agree”). The total score ranges from 9 to 63; a score of 36 or higher indicates the presence of fatigue, with higher scores indicating more severe fatigue. The scale’s overall Cronbach’s α coefficient is 0.957 [24];
4. Type D Personality Scale (DS14): The DS14 consists of 14 items across two dimensions: negative affect (6 items) and social inhibition (8 items). Responses are rated on a 5-point Likert scale, and a score of ≥ 10 on both dimensions indicates a Type D personality. The Cronbach’s α coefficient of the scale was 0.838 [25].

#### Kinesiophobia assessment tool

Tampa scale for Kinesiophobia heart (TSK-SV Heart): The TSK-SV Heart was used to assess kinesiophobia among study participants. The scale was translated into Chinese by Lei Mengjie et al. [26] and comprises four dimensions with 17 items. Total scores range from 17 to 68, with scores ≥ 37 indicating the presence of kinesiophobia. The scale demonstrated a content validity index of 0.824 and an overall Cronbach’s α coefficient of 0.859 [26].

#### General information questionnaire

A self-designed questionnaire was developed for this study. Using a combination of literature review, expert consultation, and a pilot survey, potential influencing factors were identified to construct the “General information questionnaire on kinesiophobia in postoperative cardiac surgery patients.” The questionnaire collected information on gender, age, body mass index (BMI), serum albumin, cardiac functional class, pain score, duration of mechanical ventilation, length of stay in the ICU, type of surgery, duration of cardiopulmonary bypass, history of hypertension, diabetes, history of cerebrovascular diseases, smoking history, drinking history, marital status, educational level, and monthly income.

### Data collection methods

This study collected data through a combination of face-to-face questionnaire surveys and extraction of electronic medical record information. The questionnaire surveys were conducted by investigators who had received unified training from the research team. To ensure consistency in the survey process, all investigators received standardized training before the formal survey. The training covered the study objectives and implementation procedures, obtaining informed consent, procedures for questionnaire distribution and collection, principles for using standardized instructions, non-leading explanation methods, and standards for questionnaire quality checks. To ensure the accuracy and consistency of data collection, each investigator underwent simulated data collection exercises and assessment. Only those who passed the assessment were allowed to participate in the formal survey. The survey was mainly conducted in the hospital wards during periods when patients were in relatively stable condition, the surrounding environment was quiet, and routine medical treatment and nursing care were not affected. Before the survey began, the investigators explained the study objectives, questionnaire content, and completion requirements to the patients using standardized instructions, informed them that the questionnaire results would be used only for scientific research analysis, and encouraged them to complete the questionnaire independently according to their actual situation. Completing all questionnaires usually took about 10–18 min. Self-assessment scales were, in principle, completed independently by the patients themselves. For patients who were unable to complete the questionnaire independently because of postoperative physical weakness, reading difficulties, or difficulty writing, the investigators read each questionnaire item aloud and recorded the patients’ responses accurately. If patients had questions about individual items, the investigators used only non-leading language to explain the meaning of the items or how to complete them, without providing leading hints or inducement, so as to minimize information bias as much as possible. After the questionnaires were completed, the investigators checked their completeness on site. Any missing items were completed promptly after communication with the patients. Questionnaires with obviously abnormal completion times, mechanical response patterns, numerous logical errors, substantial missing data, or non-standard completion were excluded. After all data had been collected, the principal investigators (the first author and the corresponding author) organized and verified the data. Questionnaires with a missing rate greater than 10% were excluded; a small number of missing values in the final analysis dataset were handled using multiple imputation.

### Statistical methods

Statistical analyses were conducted using SPSS 27.0 and R 4.4.3. Continuous variables with a normal distribution are presented as mean ± standard deviation ($\overline{x}\pm{s}$), and group differences were compared using the independent-samples t-test. Continuous variables not following a normal distribution are reported as median and interquartile range (M, IQR), with group comparisons performed using the Wilcoxon rank-sum test. Categorical variables are expressed as frequency and percentage, and comparisons between groups were conducted using the χ² test or appropriate nonparametric tests. The presence or absence of kinesiophobia was treated as the dependent variable. Variables that showed statistically significant differences (*P* < 0.05) in univariate analysis, along with those of clinical relevance, were included as candidate independent variables in a binary logistic regression model for multivariate analysis. To prevent potential multicollinearity from affecting model stability, the variance inflation factor (VIF) was calculated before modeling, with VIF > 5 indicating significant multicollinearity.

For the variables included in the model, we further assessed model fit and predictive performance. Internal validation was performed using the Bootstrap method with 1,000 resamples. The model’s discriminative ability was evaluated using the area under the receiver operating characteristic curve (AUC), and its calibration was assessed using the Brier score and calibration curves. The clinical utility of the model was examined through decision curve analysis (DCA), and the model was visualized as a nomogram. Statistical significance was set at α = 0.05.

## Results

### Prevalence of kinesiophobia

**Fig. 1:**
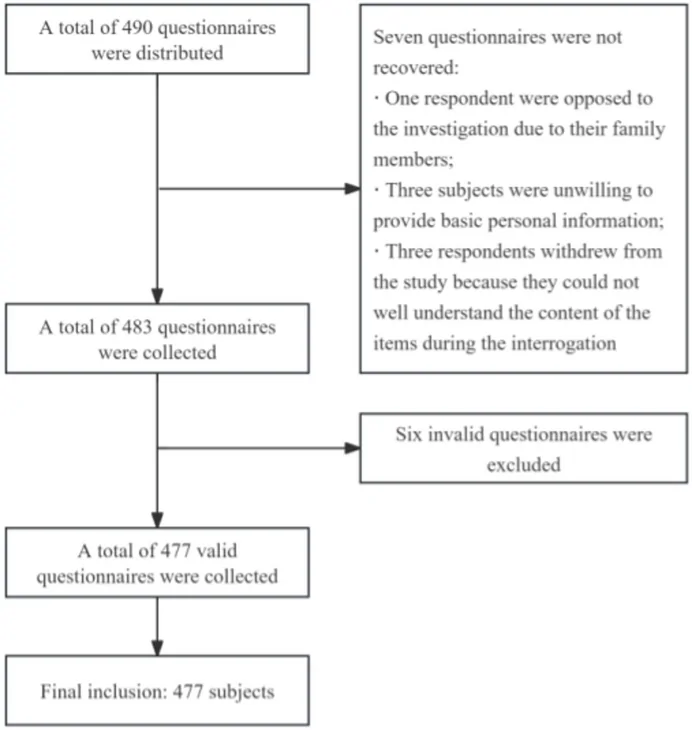
Flowchart of participant recruitment and inclusion

A total of 490 questionnaires were distributed, and 483 were returned. Six were excluded due to poor completion quality, resulting in 477 valid questionnaires, yielding an effective response rate of 97.35%. The recruitment and inclusion process is illustrated in Fig. 1. Among the participants, 292 were classified in the kinesiophobia group and 185 in the non-kinesiophobia group, corresponding to a prevalence of kinesiophobia of 61.22%.

### Comparison of clinical data between the two groups

Univariate analysis results (Table 1) indicated statistically significant differences (*P* < 0.05) between the kinesiophobia group and the non-kinesiophobia group with respect to age, cardiac function classification, level of activities of daily living, pain intensity, fatigue, Type D personality, social support level, and educational level. These findings suggest that these variables are associated with the occurrence of kinesiophobia.

**Table 1:** Comparison of clinical characteristics between the kinesiophobia and non-kinesiophobia groups (*n* = 477)

Clinical data	Kinesiophobia group	Non-kinesiophobia group	χ^2^ value	*P* value
Gender [n(%)]			1.425	0.233
Man	167(57.2%)	116(62.7%)		
Woman	125(42.8%)	69(37.3%)		
Age [n(%)]			8.403	0.015
18–44 years old	46(15.8%)	36(19.5%)		
45–59 years old	145(49.7%)	108(58.4%)		
≥ 60 years old	101(34.6%)	41(22.2%)		
BMI [n(%)]			1.442	0.486
< 18.5 kg/m^2^	35(12.0%)	16(8.6%)		
18.5 ≤ BMI < 24 kg/m^2^	180(61.6%)	121(65.4%)		
≥ 24 kg/m^2^	77(26.4%)	48(25.9%)		
Serum albumin [n(%)]			1.194	0.274
> 35 g/L	216(74.0%)	145(78.4%)		
≤ 35 g/L	76(26.0%)	40(21.6%)		
Cardiac functional class [n(%)]			31.669	< 0.001
Class Ⅰ–Ⅱ	80(27.4%)	98(53.0%)		
Class Ⅲ–Ⅳ	212(72.6%)	98(47.0%)		
Level of self-care ability [n(%)]			28.482	< 0.001
No dependence or mild dependence	95(32.5%)	106(57.3%)		
Moderate to severe dependence	197(67.5%)	79(42.7%)		
Pain intensity [n(%)]			36.746	< 0.001
No pain or mild pain	63(21.6%)	75(40.5%)		
Moderate pain	141(48.3%)	93(50.3%)		
Severe pain	88(30.1%)	17(9.2%)		
Duration of mechanical ventilation [n(%)]			0.323	0.570
< 24 h	226(77.4%)	139(75.1%)		
≥ 24 h	66(22.6%)	46(24.9%)		
Length of stay in the ICU [n(%)]			0.497	0.481
< 72 h	169(57.9%)	101(54.6%)		
≥ 72 h	123(42.1%)	84(45.4%)		
Type of surgery [n(%)]			1.958	0.581
Coronary artery bypass grafting (CABG)	97(33.2%)	51(27.6%)		
Heart valve replacement or reshaping surgery	103(35.3%)	70(37.8%)		
CABG combined with valve and artery replacement	44(15.1%)	28(15.1%)		
Others	48(16.4%)	36(19.5%)		
Duration of extracorporeal bypass [n(%)]			0.742	0.389
< 120 min	110(37.7%)	77(41.6%)		
≥ 120 min	182(62.3%)	108(58.4%)		
Hypertension [n(%)]			0.667	0.414
No	203(69.5%)	122(65.9%)		
Yes	89(30.5%)	63(34.1%)		
Diabetes [n(%)]			2.854	0.091
No	248(84.9%)	167(90.3%)		
Yes	44(15.1%)	18(9.7%)		
History of cerebrovascular diseases [n(%)]				
No	282(96.6%)	172(93.0%)	3.202	0.074
Yes	10(3.4%)	13(7.0%)		
Fatigue [n(%)]			53.107	< 0.001
No	63(21.6%)	100(54.1%)		
Yes	229(78.4%)	85(45.9%)		
Smoking history [n(%)]			1.516	0.218
No	193(66.1%)	112(60.5%)		
Yes	99(33.9%)	73(39.5%)		
Drinking history [n(%)]			1.658	0.198
No	217(74.3%)	147(79.5%)		
Yes	75(25.7%)	38(20.5%)		
Type D personality [n(%)]			29.218	< 0.001
No	106(36.3%)	114(61.6%)		
Yes	186(63.7%)	71(38.4%)		
Marital status [n(%)]			2.705	0.100
Married	260(89.0%)	173(93.5%)		
Unmarried/divorced/widowed/others	32(11.0%)	12(6.5%)		
Social support level [n(%)]			28.822	< 0.001
High level	38(13.0%)	54(29.2%)		
Moderate level	129(44.2%)	89(48.1%)		
Low level	125(42.8%)	42(22.7%)		
Educational level [n(%)]			21.478	< 0.001
College degree or above	116(39.7%)	90(48.6%)		
Middle school/High school	103(35.3%)	80(43.2%)		
Primary school and below	73(25.0%)	15(8.1%)		
Monthly income [n(%)]			4.264	0.119
> 5000 yuan	42(14.4%)	36(19.5%)		
2500–5000 yuan	127(43.5%)	87(47.0%)		
< 2500 yuan	123(42.1%)	62(33.5%)		

### Multivariate analysis

Using the presence or absence of kinesiophobia as the dependent variable, variables that were statistically significant in the univariate analysis were included in a binary logistic regression model to further identify independent factors. The variable assignments are shown in Table 2. Variables were selected based on the Akaike Information Criterion (AIC) using both forward and backward stepwise selection. The results indicated that pain intensity, social support level, fatigue, Type D personality, and educational level were independent risk factors for postoperative kinesiophobia. Severe pain was strongly associated with an increased risk of kinesiophobia (OR = 10.396, 95% CI: 4.918–21.978). Additionally, moderate pain (OR = 1.902, 95% CI: 1.151–3.142), moderate social support (OR = 2.282, 95% CI: 1.242–4.193), low social support (OR = 3.774, 95% CI: 1.894–7.518), fatigue (OR = 8.017, 95% CI: 4.773–13.468), Type D personality (OR = 3.139, 95% CI: 1.944–5.069), and an educational level of elementary school or below (OR = 3.932, 95% CI: 1.878–8.233) were also significant risk factors. Detailed results are shown in Table 3.

**Table 2:** Assignment table of independent variables

Independent variable	Assignment
Age	18–44 years old = 1; 45–59 years old = 2; ≥60 years old = 3
Cardiac functional Class	Class Ⅰ–Ⅱ = 1; Class Ⅲ–Ⅳ = 2
Level of self-care ability	No dependence or mild dependence = 1; Moderate to severe dependence = 2
Pain intensity	No or mild pain = 1; Moderate pain = 2; Severe pain = 3
Fatigue	No = 0; Yes = 1
Type D personality	No = 0; Yes = 1
Social support level	High level = 1; Moderate level = 2; Low level = 3
Educational level	College degree or above = 1; Middle School/High School = 2; Primary school and below = 3

**Table 3:** Results of logistic regression analysis

Variables	β value	SE value	Wald χ^2^ value	*P* value	OR value	95% CI
Pain intensity (with no or mild pain as the reference)			37.622	**< 0.001**		
Moderate pain	0.643	0.256	6.293	0.012	1.902	1.151–3.142
Severe pain	2.341	0.382	37.582	**< 0.001**	10.396	4.918–21.978
Social support level (with the high level serving as the reference point)			14.297	**< 0.001**		
Moderate level	0.825	0.310	7.064	0.008	2.282	1.242–4.193
Low level	1.328	0.352	14.260	**< 0.001**	3.774	1.894–7.518
Fatigue	2.082	0.265	61.865	**< 0.001**	8.017	4.773–13.468
Type D personality	1.144	0.244	21.899	**< 0.001**	3.139	1.944–5.069
Educational level (Based on a college degree or higher level)			21.983	< **0.001**		
Middle School/High School	-0.465	0.250	3.452	0.063	0.628	0.385–1.026
Primary school and below	1.369	0.377	13.187	**< 0.001**	3.932	1.878–8.233
Constant	-3.024	0.419	51.978	**< 0.001**	0.049	

Note: Bold values indicate statistically significant differences at *P* < 0.001

### Diagnosis of multicollinearity

Multicollinearity among the five variables was assessed using VIF. The results (Table 4) indicated that the collinearity tolerance for pain intensity, fatigue, Type D personality, social support level, and educational level were all greater than 0.1, and the corresponding VIF values were all below 5. These findings suggest that there is no significant multicollinearity among the covariates, and all variables can be included in the predictive model.

**Table 4:** Collinearity diagnostics of predictor variables

Variables	Collinearity tolerance	Variance inflation factor
Pain intensity	0.972	1.029
Fatigue	0.994	1.006
Type D personality	0.928	1.077
Social support level	0.905	1.105
Educational level	0.943	1.060

### Nomogram model

**Fig. 2:**
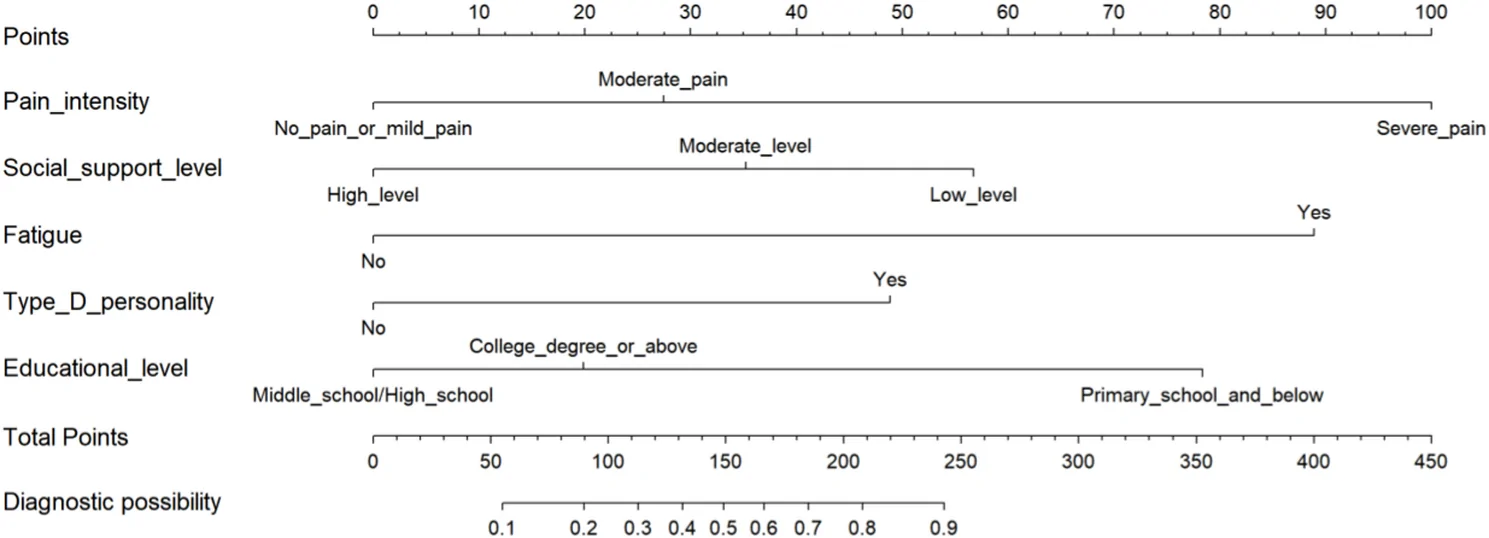
Nomogram model for predicting the risk of kinesiophobia in post-Cardiac surgery patients

Prior to modeling, the outcome variable was dichotomously coded. Data distribution and model fit were examined, and no complete separation or significant outliers were observed that could compromise model stability. Most variables included in the study were categorical, while continuous variables were grouped according to clinical relevance before modeling. Multicollinearity was assessed using VIF to ensure model stability. Ultimately, a nomogram model for predicting the risk of kinesiophobia in post-cardiac surgery patients was developed based on five variables: pain intensity, fatigue, Type D personality, social support level, and educational level (Fig. 2). The model allows calculation of a total score by summing the points assigned to each predictor variable, which can then be converted into an individual’s probability of developing kinesiophobia. Application method: For each predictor variable, a vertical line is drawn from the patient’s value to the points axis to obtain the corresponding score. This procedure is repeated for all predictors, and the individual scores are summed. The total score is then mapped to the probability scale to estimate the patient’s risk of kinesiophobia.

**Fig. 3:**
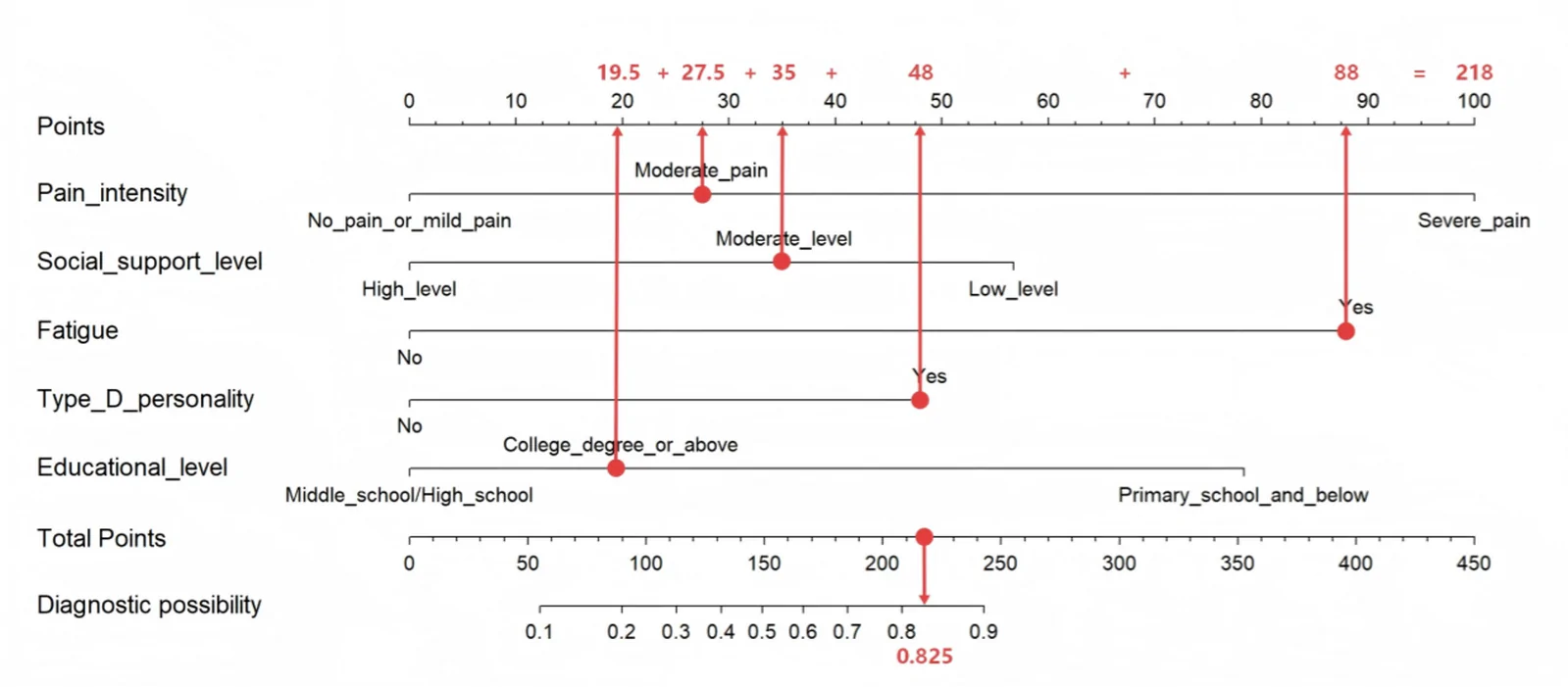
Example of nomogram application for predicting the risk of kinesiophobia

Example of nomogram application: A post-cardiac surgery patient presented with moderate pain, moderate social support, Type D personality, fatigue, and a college-level education. Based on these characteristics, the nomogram was used to estimate the probability of developing kinesiophobia. The detailed procedure is illustrated in Fig. 3. (1) Pain intensity: “Moderate pain” was located on the corresponding axis, and a vertical line was drawn upward to the Points axis, yielding a score of 27.5 points. (2) Social support level: “Moderate level” was identified on the corresponding axis, and a vertical line was drawn upward to the Points axis, yielding a score of 35 points. (3) Fatigue: The patient was classified as having fatigue, corresponding to a score of 88 points on the Points axis. (4) Type D personality: The “Yes” category was selected, corresponding to a score of 48 points. (5) Educational level: “College degree or above” was identified on the corresponding axis, corresponding to a score of 19.5 points. The scores for the five predictors were summed to obtain a total score of 218 points. On the Total Points axis of the nomogram, a vertical line was drawn downward from 218 to the Predict Value axis, corresponding to an estimated probability of approximately 0.825. Therefore, the predicted probability of kinesiophobia in this patient was approximately 82.5%.

### Model performance evaluation

**Fig. 4:**
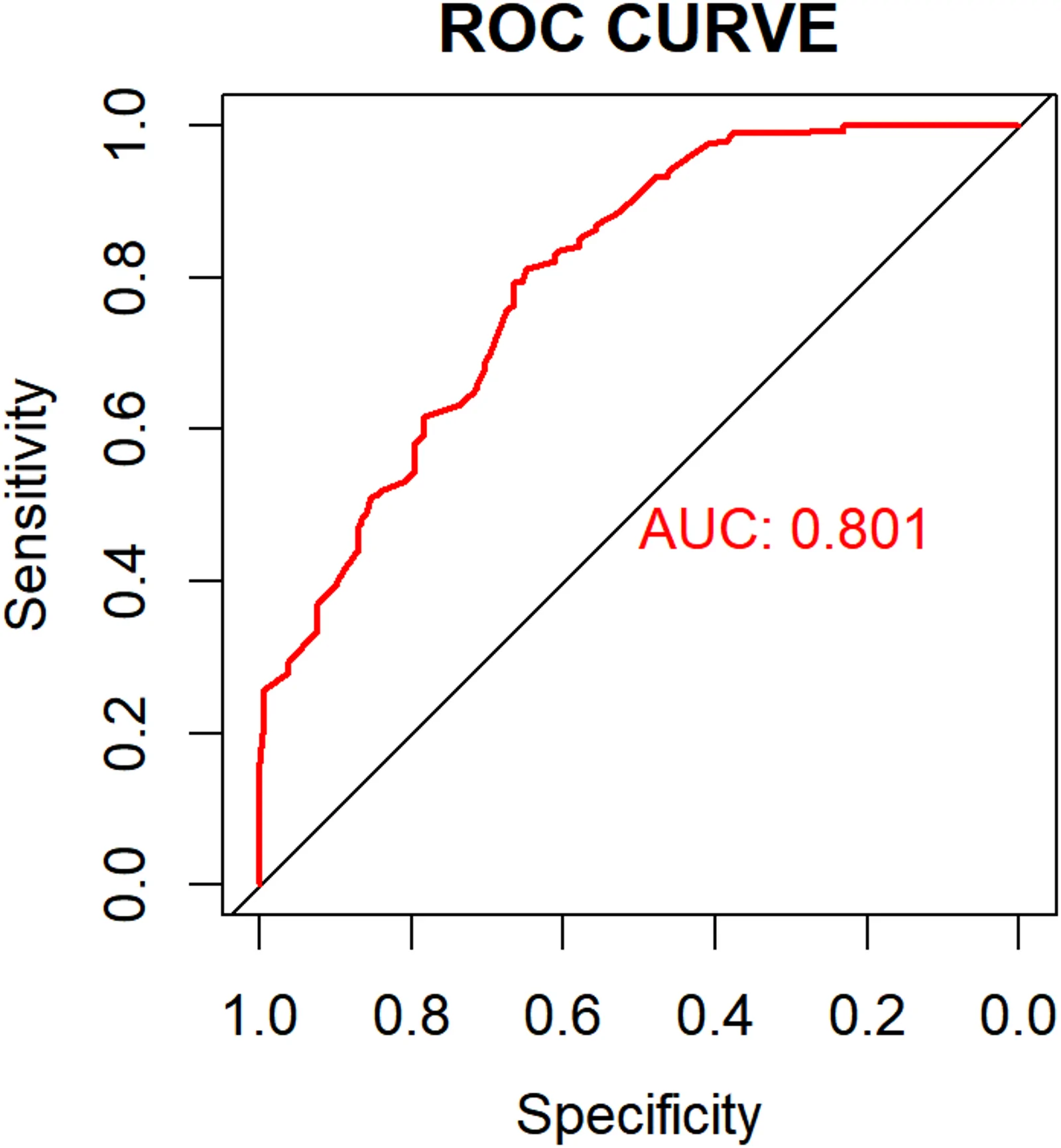
Receiver operating characteristic (ROC) curve

**Fig. 5:**
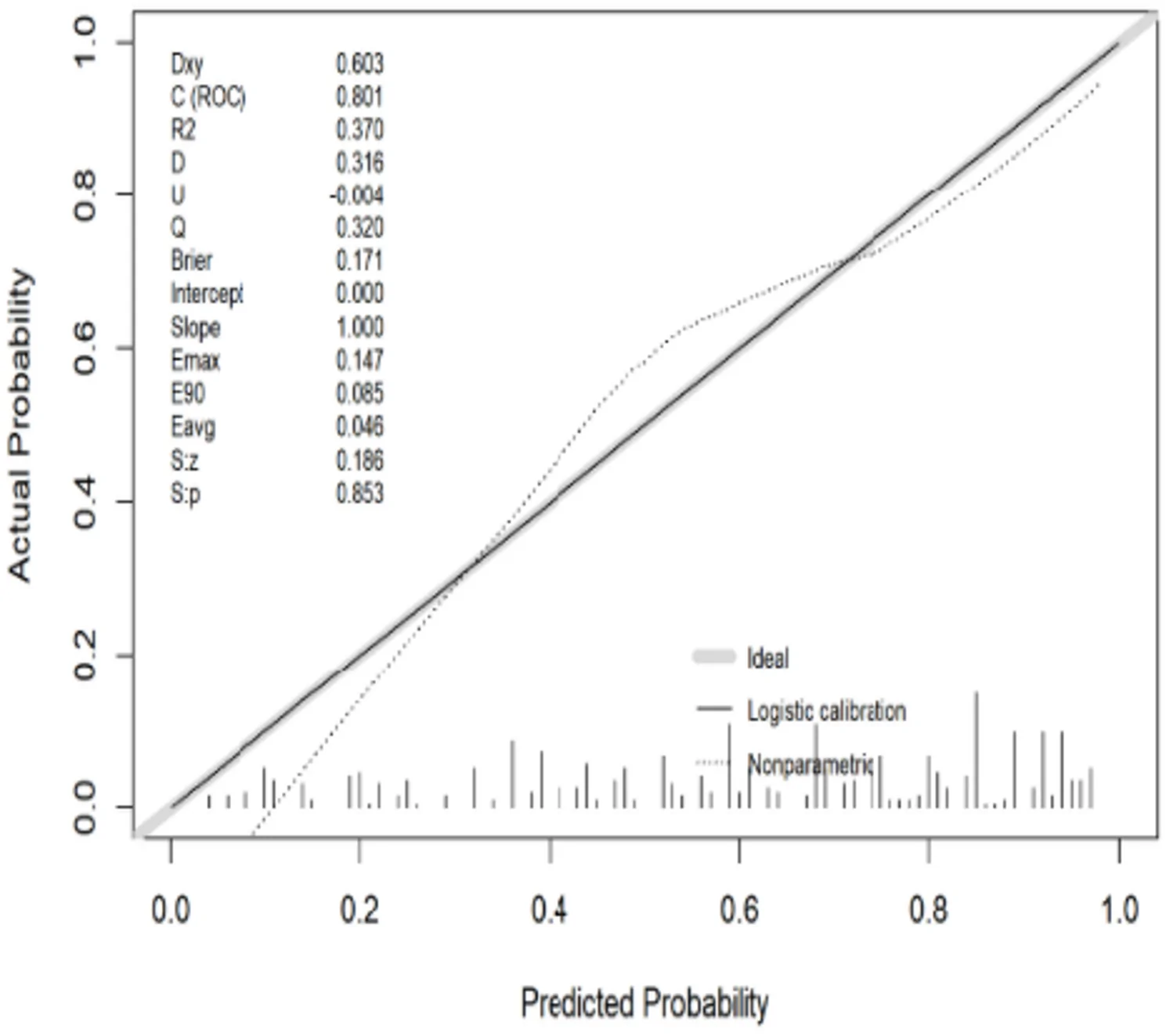
Calibration curve

**Fig. 6:**
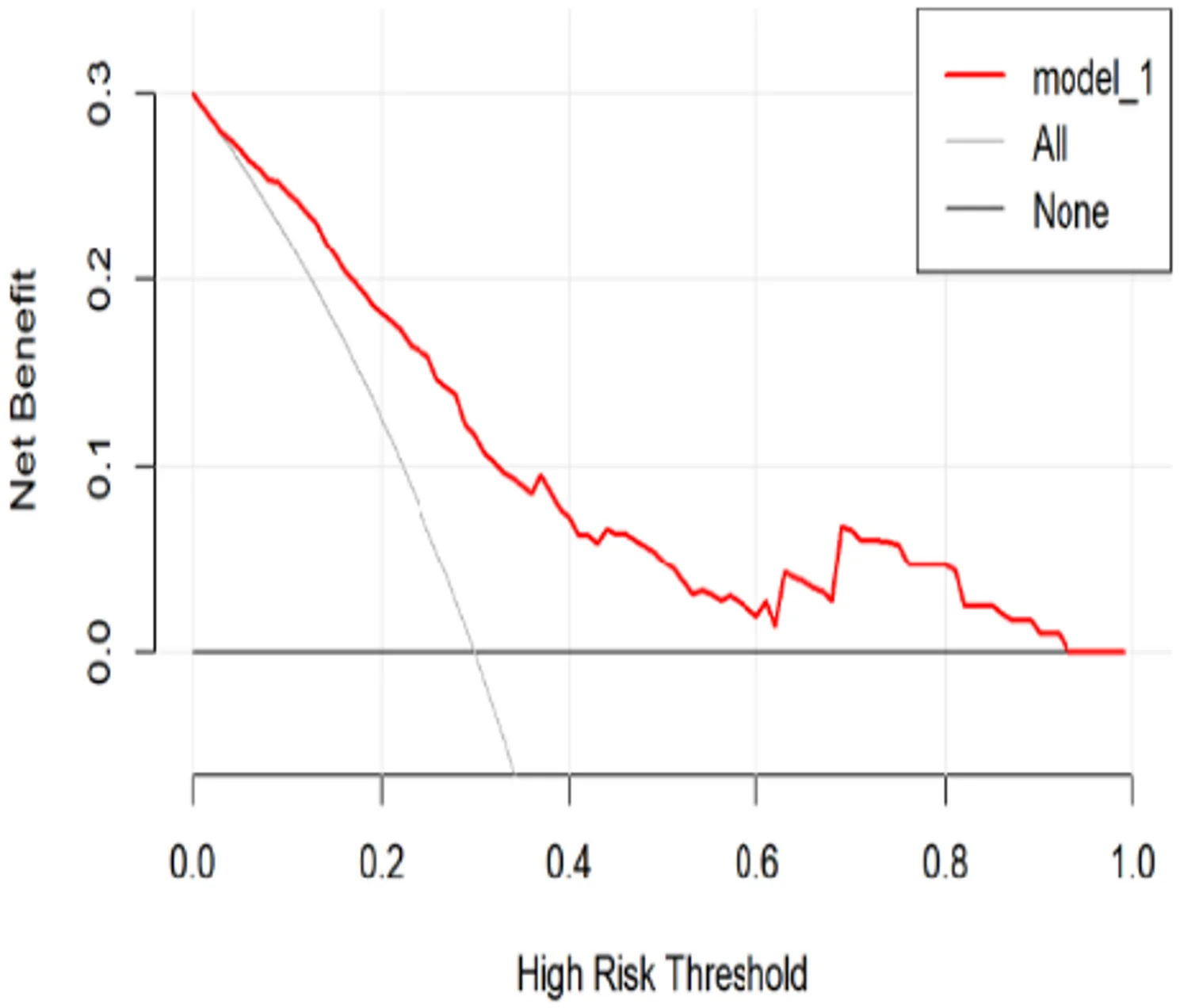
Decision curve analysis (DCA)

Internal validation of the model was performed using the Bootstrap method with 1,000 resamples. Model performance was evaluated in terms of discrimination, calibration, and clinical utility. Discriminative ability was assessed using AUC. As shown in Fig. 4, the model achieved an AUC of 0.801 (95% CI: 0.761–0.842), indicating good discrimination between patients with and without kinesiophobia. Calibration was evaluated using the Brier score and calibration curves. The Brier score was 0.171 (95% CI: 0.153–0.189), indicating a relatively small discrepancy between the predicted probabilities and the observed outcomes. The calibration curve (Fig. 5) demonstrated good agreement between the predicted and observed probabilities, suggesting satisfactory calibration performance of the model. In addition, clinical utility was assessed using DCA. As shown in Fig. 6, when the threshold probability ranged from 0.02 to 0.92, use of the model for clinical decision-making provided a substantial net benefit, indicating good clinical applicability.

## Discussion

### Analysis of the current status

This study included a total of 477 post-cardiac surgery patients, among whom 292 developed kinesiophobia, yielding a prevalence rate of 61.22%. A review of previous studies indicated that this finding is highly consistent with the prevalence range of kinesiophobia reported in multiple studies [12, 27] involving post-cardiac surgery patients, further confirming the widespread occurrence of kinesiophobia following cardiac surgery.

Previous studies have demonstrated that fear of exercise not only reduces adherence to cardiac rehabilitation [28] but is also closely associated with increased levels of anxiety and depression [29, 30], which may further aggravate cardiac dysfunction [13, 14]. Differences in prevalence rates reported across studies may be attributable to variations in surgical type, perceptions of exercise, and timing of assessment. Nevertheless, the findings of the present study suggest that more than half of post-cardiac surgery patients experience kinesiophobia.

Although increasing attention has been directed toward kinesiophobia in recent years, its prevalence has not shown a declining trend. Therefore, healthcare professionals should fully recognize the importance of managing kinesiophobia in post-cardiac surgery patients and conduct comprehensive and systematic assessments at an early stage to promptly identify high-risk individuals. In addition, greater emphasis should be placed on the management of high-risk patients and related influencing factors. For patients who have already developed kinesiophobia, individualized interventions should be actively implemented to help them gradually overcome fear, thereby promoting participation in exercise rehabilitation and reducing adverse health outcomes.

### Analysis of influencing factors

The findings of this study indicate that moderate-to-severe pain is an important influencing factor for kinesiophobia in post-cardiac surgery patients, which is consistent with previous studies suggesting that pain may increase exercise avoidance behavior [31]. Patients experiencing severe postoperative pain after cardiac surgery are generally more sensitive to potential pain-related cues in their environment, leading to excessive attention to pain itself and to stimuli associated with pain, ultimately contributing to the development of kinesiophobia [32]. In addition, prolonged activity restriction caused by pain may result in muscle atrophy and joint stiffness, further reducing exercise tolerance. Consequently, patients are more likely to experience discomfort when attempting physical activity, which may in turn exacerbate pain perception and kinesiophobia [33], thereby creating a vicious cycle. These findings suggest that patients with more severe postoperative pain are at a higher risk of developing kinesiophobia after cardiac surgery. It should be noted that although analgesics may temporarily relieve pain, long-term use should be avoided [34] because of the potential risk of drug dependence and interference with rehabilitation training. Therefore, postoperative pain management, pain education, and staged activity guidance should be integrated with rehabilitation training to reduce pain-related kinesiophobia.

Fatigue was another factor closely associated with kinesiophobia in this study. Following cardiac surgery, patients often experience persistent fatigue due to inflammatory responses, poor sleep quality, and inadequate recovery of cardiopulmonary function [35]. Previous studies have also demonstrated that [36] fatigue is one of the major barriers limiting participation in exercise-based rehabilitation among patients with cardiovascular disease. Patients experiencing fatigue generally have insufficient physical reserves, and even mild physical activity may easily induce discomfort such as dyspnea and weakness. Consequently, patients may develop negative perceptions [37] that they are unable to tolerate exercise, thereby reducing exercise self-efficacy and increasing avoidance behaviors. In addition, fatigue may impair emotional regulation, making patients more susceptible to feelings of helplessness and anxiety, which may further intensify fear of exercise [36]. Therefore, fatigue screening should be incorporated into routine postoperative rehabilitation assessment. In addition, graded activity strategies should be developed in combination with exercise prescription, sleep management, and nutritional support. Low-intensity, high-frequency exercise interventions may help patients gradually build confidence, improve physical function, and reduce fatigue-related kinesiophobia.

Type D personality was identified as one of the major psychological risk factors in this study and is primarily characterized by negative affectivity and social inhibition. Patients with Type D personality are more likely to experience anxiety and depression when coping with illness, exhibit heightened sensitivity to postoperative discomfort, and tend to suppress negative emotions. In addition, they are less likely to actively express distress or seek social support and may be reluctant to participate in rehabilitation exercise programs [38, 39]. Previous studies in the cardiovascular field have demonstrated that [40, 41] Type D personality is closely associated with poorer psychological adaptation and lower participation in rehabilitation. Therefore, early identification of personality characteristics in post-cardiac surgery patients and timely implementation of targeted psychological interventions are particularly important. Healthcare professionals may incorporate psychological strategies such as cognitive behavioral therapy, mindfulness-based interventions, and peer support to help correct maladaptive cognitive patterns, reduce anxiety levels, and minimize the influence of negative thoughts on exercise-related decision-making.

The level of social support was also identified as an important influencing factor in this study. Social support not only provides emotional comfort for patients but also helps them develop a sense of security regarding rehabilitation exercise through informational support and behavioral modeling. In contrast, patients lacking family and peer support are more likely to experience feelings of loneliness and helplessness when facing postoperative discomfort. Their concerns about exercise-related risks may not be alleviated through communication [42], and without encouragement from family members or others, they may be more likely to abandon attempts at physical activity. Previous studies have also suggested that family involvement and positive caregiving support can improve rehabilitation behaviors and health outcomes among patients with cardiovascular disease [43]. Therefore, it is recommended that healthcare professionals incorporate family education into discharge planning and follow-up management. Family members should be guided to provide emotional support through accompanying patients during exercise and offering positive encouragement. In addition, postoperative rehabilitation peer-support groups may be established, allowing patients with favorable rehabilitation outcomes to share their experiences and help individuals with low or moderate levels of social support build social connections. By providing comprehensive emotional support and multiple sources of health-related information, a supportive environment conducive to exercise rehabilitation may be fostered.

The findings of this study indicate that patients with an educational attainment of elementary school or below were more likely to develop kinesiophobia, which is consistent with previous studies [44]. Patients with lower educational levels generally possess less medical knowledge and have a reduced ability to understand health-related information, which may result in insufficient disease cognition and negatively affect adherence to exercise rehabilitation [45, 46]. For this population, postoperative exercise education may be delivered through accessible approaches such as social media platforms, public health accounts, and short educational videos to provide convenient informational support. In addition, educational sessions related to exercise rehabilitation may also be organized. Strengthening patient health education can help improve patients’ understanding of the benefits of exercise, thereby alleviating fear related to physical activity and promoting participation in rehabilitation exercise.

### Analysis of model performance

The nomogram model developed in this study effectively predicts the risk of kinesiophobia in patients following cardiac surgery. It is characterized by simplicity, cost-effectiveness, and practicality, and enables individualized risk assessment through visualization. Compared to single-factor risk analysis, it is better suited for rapid clinical decision-making and aids in the early screening of patients after cardiac surgery. Furthermore, following internal validation, the model demonstrated good discriminatory power, calibration, and clinical net benefit, making it suitable for accurately predicting the risk of kinesophobia in patients following cardiac surgery. Additionally, the five risk factors incorporated into the model developed in this study integrate variables from the bio-psycho-social dimensions and are readily available, further enhancing the model’s clinical accessibility. This ensures the smooth implementation of the predictive model in clinical practice and underscores its strong practicality.

In clinical practice, this model may be applied to early postoperative rehabilitation assessment, pre-discharge risk stratification, and follow-up management in patients undergoing cardiac surgery. Healthcare professionals can estimate the risk of kinesiophobia based on patients’ pain intensity, fatigue level, personality characteristics, social support level, and educational level. According to the assessed risk level, more targeted interventions may be implemented for high-risk patients, including optimization of pain management strategies, reinforcement of exercise education, provision of psychological support, involvement of family members in rehabilitation guidance, and development of individualized exercise prescriptions. For low-risk patients, rehabilitation may proceed according to standard rehabilitation protocols. Overall, the predictive model developed in this study may improve the efficiency of healthcare resource utilization, enhance adherence and safety in exercise rehabilitation, and reduce the occurrence of kinesiophobia among post-cardiac surgery patients.

### Analysis of innovations and limitations

The innovations of this study are primarily reflected in the following aspects: (1) This study focused specifically on post-cardiac surgery patients, a population characterized by distinct disease-specific features and unique rehabilitation needs, rather than general postoperative populations; (2) Factors associated with kinesiophobia were identified and analyzed from multiple dimensions, including biological, psychological, and social support factors, thereby enhancing the clinical interpretability of the selected variables; (3) A nomogram prediction model was developed and internally validated using the bootstrap method, enabling visualization and individualized assessment of risk; (4) Targeted nursing and rehabilitation intervention strategies were proposed based on the model results, facilitating the translation of research findings from risk prediction into clinical practice. Nonetheless, this study has five key limitations. First, due to the cross-sectional study design, this study could only identify associations between kinesiophobia and potential risk factors among post-cardiac surgery patients at the time of investigation, and causal relationships could not be established. Future studies adopting prospective longitudinal designs are warranted to further clarify the dynamic interactions among these variables over time. Second, all participants were recruited from a single Grade A tertiary hospital in Shanghai. Differences in regional healthcare resources, perioperative management models, and demographic characteristics may limit the representativeness of the sample and the generalizability of the findings. Therefore, caution should be exercised when extrapolating the results to other regions or healthcare settings. Third, several key variables were collected primarily through self-reported questionnaires, which may be influenced by recall bias and participants’ emotional states during assessment, potentially resulting in information bias or measurement error. Future studies may incorporate objective clinical indicators and multimodal assessment approaches to improve measurement accuracy and data reliability. In addition, postoperative duration, a potential influencing factor, was not included in the variable collection of this study, which may have affected the explanatory power of the model to some extent. Therefore, further validation using longitudinal studies with multiple postoperative time points is warranted. Finally, the model was validated only internally through Bootstrap resampling. Although the results demonstrated good discrimination and calibration performance, external validation using independent datasets has not yet been performed. Therefore, the generalizability and clinical applicability of the model across different regions, institutions, and temporal cohorts require further evaluation. Future studies may conduct multicenter external validation and incorporate machine learning approaches to further optimize the model, thereby improving its stability and clinical utility.

## Conclusions

This study developed a nomogram model incorporating pain intensity, fatigue, Type D personality, social support level, and educational level, which demonstrated good performance in predicting the risk of kinesiophobia among post-cardiac surgery patients. By applying this predictive model, healthcare professionals can identify high-risk individuals at an early stage, thereby providing a scientific basis for the development of individualized health education strategies and the optimization of exercise rehabilitation programs.

## Supplementary Information

Supplementary Material 1.

## Data Availability

The original contributions presented in the study are included in the article/supplementary material, further inquiries can be directed to the corresponding authors.

## Abbreviations

AUC: Area under the receiver operating characteristic curve
BI: Barthel Index
BMI: Body mass index
CVD: Cardiovascular disease
CI: Confidence interval
DCA: Decision curve analysis
DS14: Type D Personality Scale
FSS: Fatigue Severity Scale
ROC curve: receiver operating characteristic curve
SSRS: Social Support Rating Scale
TSK-SV Heart: Tampa scale for Kinesiophobia heart
VIF: Variance inflation factor
