# Supplementary material for: Analysis of factors influencing kinesiophobia in post-cardiac surgery patients and construction of a nomogram prediction model
Source: BMC Cardiovasc Disord. 2026 Aug 1;26:994. doi: 10.1186/s12872-026-06301-x (PMC13721678; doi:10.1186/s12872-026-06301-x)
Supplement: Supplementary file 1 [file 12872_2026_6301_MOESM1_ESM.docx]

**General Information Questionnaire**

**Living and working conditions layer**

1.Educational level: □College degree or above □Middle school/High school □Primary school and below

2.Monthly income: □<2500 yuan □2500–5000 yuan □>5000 yuan

**Interpersonal relationship layer**

1.Marital status: □Married □Unmarried/divorced/widowed/others

2.Social support level: □Low level □Moderate level □High level

**Behavioral and psychological levels**

1.Type D personality: □Yes □No

2.Smoking history: □Yes □No

3.Drinking history： □Yes □No

**Personal traits layer:**

1.Gender: □Man □Woman

2.Age: □18–44 years old □45–59 years old □≥60 years old

3.BMI: □<18.5 kg/m2 □18.5≤BMI<24 kg/m2 □≥24 kg/m2

4.Type of surgery: □ Coronary artery bypass grafting (CABG) □ Heart valve replacement or reshaping surgery □ CABG combined with valve and artery replacement □ Others

5.Duration of extracorporeal circulation: □ <120 minutes □ ≥120 minutes

6.Serum albumin: □ >35 g/L □ ≤35 g/L

7.Level of self-care ability: □No dependence or mild dependence □Moderate to severe dependence

8.Pain intensity: □No pain or mild pain □Moderate pain □Severe pain

9.Duration of mechanical ventilation: □<24 hours □≥24 hours

10.Cardiac functional grading: □ Grade Ⅰ–Ⅱ □ Grade Ⅲ–Ⅳ

11.Length of stay in the ICU: □<72 hours □≥72 hours

12.Hypertension: □Yes □No

13.Diabetes: □Yes □No

14.History of cerebrovascular diseases: □Yes □No

15.Fatigue: □Yes □No
